# Integrated proteomic and metabolomic profile analyses of cardiac valves revealed molecular mechanisms and targets in calcific aortic valve disease

**DOI:** 10.3389/fcvm.2022.944521

**Published:** 2022-10-13

**Authors:** Bo Fu, Jing Wang, Lianqun Wang, Qiang Wang, Zhigang Guo, Meilin Xu, Nan Jiang

**Affiliations:** ^1^Department of Cardiovascular Surgery, Tianjin Chest Hospital, Tianjin, China; ^2^Postdoctoral Mobile Station, Tianjin Medical University, Tianjin, China; ^3^Department of Pathology, Tianjin Chest Hospital, Tianjin, China

**Keywords:** calcific aortic valve disease, proteomics, metabolomics, cardiac valve, molecular

## Abstract

**Background:**

This study aimed to define changes in the metabolic and protein profiles of patients with calcific aortic valve disease (CAVD).

**Methods and results:**

We analyzed cardiac valve samples of patients with and without (control) CAVD (*n* = 24 per group) using untargeted metabolomics and tandem mass tag-based quantitative proteomics. Significantly different metabolites and proteins between the CAVD and control groups were screened; then, functional enrichment was analyzed. We analyzed co-expressed differential metabolites and proteins, and constructed a metabolite-protein-pathway network. The expression of key proteins was validated using western blotting. Differential analysis identified 229 metabolites in CAVD among which, 2-aminophenol, hydroxykynurenine, erythritol, carnosine, and choline were the top five. Proteomic analysis identified 549 differentially expressed proteins in CAVD, most of which were localized in the nuclear, cytoplasmic, extracellular, and plasma membranes. Levels of selenium binding protein 1 (SELENBP1) positively correlated with multiple metabolites. Adenosine triphosphate-binding cassette transporters, starch and sucrose metabolism, hypoxia-inducible factor 1 (HIF-1) signaling, and purine metabolism were key pathways in the network. Ectonucleotide pyrophosphatase/phosphodiesterase 1 (ENPP1), calcium^2+^/calmodulin-dependent protein kinase II delta (CAMK2D), and ATP binding cassette subfamily a member 8 (ABCA8) were identified as hub proteins in the metabolite-protein-pathway network as they interacted with ADP, glucose 6-phosphate, choline, and other proteins. Western blotting confirmed that ENPP1 was upregulated, whereas ABCA8 and CAMK2D were downregulated in CAVD samples.

**Conclusion:**

The metabolic and protein profiles of cardiac valves from patients with CAVD significantly changed. The present findings provide a holistic view of the molecular mechanisms underlying CAVD that may lead to the development of novel diagnostic biomarkers and therapeutic targets to treat CAVD.

## Introduction

Calcific aortic valve disease (CAVD) is the most prevalent heart valvular disease among elderly persons. Although CAVD has various causes, aortic valve calcification is the main pathological change (1). The amount of newly diagnosed CAVD globally increased 3.51-fold between 1990 and 2019 (2). CAVD progresses from mild calcification of the valve leaflets such as in aortic valve sclerosis to severe calcification such as aortic stenosis (AS) with hemodynamic instability (3). It has traditionally been considered a degenerative condition of the aortic valve, accumulating clinical and histopathological evidence indicates that it is an active condition involving lipid deposition, chronic inflammation, osteogenesis of valvular interstitial cells, and lobular calcification (4–6). No medical treatment has yet been found that can prevent or reduce CAVD progression; however, aortic valve replacement (AVR) and transcatheter aortic valve implantation (TAVI) can alleviate symptoms and improve prognosis (7, 8).

Although early drug intervention might be more effective in preventing disease progression (9), early valvular heart disease is asymptomatic and progresses slowly. Therefore, new biomarkers are required to improve the accuracy of early diagnosis or targets for intervention in the early stages. Investigations into molecular disease mechanisms using genomics, transcriptomics, proteomics and metabolomics have led to the discovery of potential diagnostic and prognostic biomarkers or therapeutic targets for CAVD (10–14). Obvious changes in metabolites in the coronary sinus, peripheral vein, and ascending aorta within 30 min of TAVI have been identified based on metabolic profiles of 59 patients with AS (15). The study also revealed associations between left ventricle regression and metabolites of proline and arginine. A similar study found that the metabolite acylcarnitine correlated significantly and negatively with the left ventricular ejection fraction but positively with the brain natriuretic peptide, and that phosphatidylcholines correlated significantly and negatively with the left ventricular mass (16). Proteomic profiles of 20 patients with AS identified several significantly altered proteins in aortic valves compared with normal controls, and that they are essential for coagulation, fibrosis, homeostasis, and other cardiovascular processes (17). Significantly changed protein expression in the atrial and ventricular myocardium plays important roles in AS; for example, by promoting fibrosis, and cardiac hypertrophy, and decreasing the blood supply (18).

However, the findings of a single study are insufficient to fully understand the complexity of the CAVD (19). Therefore, different types of omics data should be integrated into a biologically relevant context of the disease to elucidate interactions across disease layers and establish deeper molecular networks (20). Here, we analyzed integrated metabolomic and proteomic profiles of changes in proteins and metabolites in cardiac valve samples from patients with CAVD and controls. Our findings provide potential targets and a foundation for further investigation of CAVD.

## Materials and methods

### Patients and sample collection

Cardiac valve samples, excluding the bicuspid aortic valve and rheumatic heart disease, were collected from 24 patients after surgical aortic valve replacement for severe AS. Symptomatic patients with no-calcified severe aortic regurgitation were collected as controls (*n* = 24). The Medical Ethics Committee at our hospital approved the study (IRB protocol number: IRB-SOP-016F-001-02-2022LW-008), and all patients who were recruited from the same institution provided written, informed consent to participate. Table 1 shows the demographics, medical history, metabolic data and preoperative medication provided to the participants. Other than age and diabetes history, the clinical information did not significantly differ between the two groups.

**TABLE 1:** Clinical characteristics of calcific aortic valve disease (CAVD) patients and control individuals.

	CAVD (*n* = 24)	Control (*n* = 24)	*P*-value
Age (years)	66.5 ± 7.8	59.5 ± 12.0	<0.05
Male, n (%)	12, (50.0%)	14, (58.3%)	0.56
Body mass index	23.3 ± 3.0	23.0 ± 3.7	0.85
LVEF,%	55.5 ± 8.0	52.3 ± 9.8	0.25
**Medical history, n (%)**			
Hypotension	11, (45.8%)	16, (66.7%)	0.24
Diabetes	5, (20.8%)	0, (0%)	<0.05
CHD	15, (62.5%)	9, (37.5%)	0.08
**Preoperative Medication, n (%)**			
ACEI	1, (4.1%)	5, (20.8%)	0.08
BB	18, (75.0%)	14, (63.6%)	0.40
CCB	8, (33.3%)	5, (20.8%)	0.33
**Metabolic data**			
HDL (mmol/L)	1.04 ± 0.36	1.09 ± 0.23	0.64
LDL (mmol/L)	2.89 ± 1.17	2.36 ± 0.67	0.07
VLDL (mmol/L)	0.21 (0.20)	0.29 (0.24)	0.26
Triglyceride (mmol/L)	1.02 (0.59)	1.25 (1.07)	0.44
Lipoprotein A (nmol/L)	37.40 (50.25)	45.25 (58.20)	0.89
Apolipoprotein A (g/L)	1.22 ± 0.24	1.25 ± 0.18	0.65
Apolipoprotein B (g/L)	0.99 ± 0.29	0.83 ± 0.20	0.04

Data was presented as mean ± standard deviation or n (%) or median (interquartile range); LVEF, left ventricular ejection fraction; CHD, congenital heart disease; ACEI, angiotensin-converting enzyme inhibitors; BB, beta-blocker; CCB, calcium channel blockers; HDL, high-density lipoprotein; LDL, low-density lipoprotein; VLDL, very low-density lipoprotein.

### Immunohistochemical staining

Paraffin-embedded cardiac valve tissue sections were deparaffinized, rehydrated and immersed in citrate buffer for antigen retrieval. Endogenous peroxidases and non-specific binding were sequentially blocked using 3% H_2_O_2_ followed by bovine serum albumin, respectively. The sections were then incubated with anti-osteocalcin (OCN, catalog no. sc-365797, mouse monoclonal IgG_3_ κ, Santa Cruz, CA, United States), anti-osteopontin (OPN, catalog no. ab8448, rabbit polyclonal, Abcam, United States), and anti-runt-related transcription factor 2 (RUNX2, catalog no. ab76956, mouse monoclonal, c, United States) primary antibodies at 4°C overnight, followed by HRP-labeled secondary antibody for 50 min. The sections were then incubated with diaminobenzidine for 2 h at 37°C, counterstained with hematoxylin, rinsed, air-dried, sealed with neutral resin and examined using fluorescence microscopy.

### Histological assessment of cardiac valve calcification and collage fibers

Calcium deposition and collagen fibers in deparaffinized, paraffin-embedded tissue sections were respectively quantified using von Kossa and hematoxylin-eosin stains. The sections were then irradiated with an ultraviolet lamp for 4 h, and visualized using light microscopy.

### Untargeted metabolomic analysis

#### Metabolite extraction

Cardiac valve samples (100 mg) were pulverized in 1 mL of tissue extract comprising 25% H_2_O and 75% methanol: chloroform (9:1) at 50 Hz for 60 s using a SCIENTZ-48 high-throughput tissue grinder (SCIENTZ Biotechnology Co., Ltd., Ningbo, China). Ground tissue samples were ultrasonicated at 37°C for 30 min, followed by centrifugation at 12,000 rpm for 10 min. The supernatant was vacuum-dried, and dissolved in 200 μL 2-chlorobenzalanine (4 ppm) in 50% acetonitrile, and filtered through a PTFE membrane (0.22-μm pores). A quality control (QC) sample was prepared by mixing 20 μL of each sample to monitor deviations in the analytical results, and the remaining samples were analyzed using LC–MS as described below.

#### LC–MS analysis

Analytes were eluted through an ACQUITY UPLC^®^ HSS T3 column 150 × 2.1 mm, 1.8 μm (Waters Corp., Milford, MA, United States) gradient at a flow rate of 0.25 mL/min under autosampler and column temperatures of 8°C and 40°C, respectively. The mobile phase comprised 0.1% formic acid water (A1) in 0.1% formic acid acetonitrile (B1) or 5 mM c (A2) in acetonitrile (B2), and the gradient elution conditions were 2% B1/B2 0–1 min, 2%–50% B1/B2 1–9 min, 50%–98% B1/B2 9–12 min, 98% B1/B2 12–13.5 min, 98%–2% B1/B2 13.5–14 min, and 2% B1 14–20 min in positive ion mode, or 2% B2 14–17 min in negative ion mode The spray voltage was 3.5 or −2.5 kV in positive or negative ion modes, respectively, for MS electrospray ionization. The conditions were set as: sheath gas, 30 arb; auxiliary gas, 10 arb; capillary temperature, 325°C. The analyzer scanned over a mass range of m/z 81–1,000 for a full scan at a mass resolution of 60,000.

#### Data processing and analysis

Raw data were converted into mzXML format, and peaks were identified, filtered, aligned, and batch-normalized using the XCMS package in R 3.3.2. A data matrix, comprising the mass:charge ratio (m/z), retention time, and mass spectrum response intensity (peak area) was determined. Multivariable statistics were analyzed using the Ropls package in R, including principal component analysis (PCA), partial least squares discriminant analysis (PLS-DA), and orthogonal projections to latent structures discriminant analysis (OPLS-DA).

Overfitting the PLS-DA model was evaluated using a permutation plot. Based on the variable importance for the projection (VIP) value calculated in the OPLS-DA model, significant differential metabolites were selected with VIP ≥ 1 and *P* < 0.05. Metabolite structures were identified using the accurate molecular weight (error < 30 PPM), followed by matching to the Human Metabolome Database, Massbank, LipidMaps, Metlin, Mzcloud, and the Panomik standard databases. Differential Kyoto Encyclopedia of Genes and Genomes (KEGG) metabolic pathways were screened based on the MetPA database with a hypergeometric test, and a cut-off of *P* < 0.05, to determine significant differential KEGG metabolic pathways.

### Proteomic analysis

#### Protein extraction, digestion, and tandem mass tag labeling

Cardiac valve tissues were lysed in 100 mM Tris−HCl containing 1 mM dithiothreitol and 4% sodium-dodecyl-sulfate final pH 7.6, and proteins were extracted and quantified using BCA Protein Assay kits. Proteins were digested with trypsin using the FASP method (21). Digested peptides were desalted using C18 cartridges (Empore™ SPE, bed internal diameter, 7 mm; volume, 3 mL) (Sigma-Aldrich Corp., St. Louis, MO, United States), vacuum-dried, and reconstituted in 40 μL of 0.1% (v/v) formic acid. The peptide mixture was then labeled with the tandem mass tag (TMT) reagent as described by the manufacturer.

#### Fractionation and LC–MS/MS analysis

Peptides labeled with TMT were separated using a high-pH reversed-phase peptide fractionation kit. Bound peptides were eluted into 10 fractions, collected using vacuum centrifugation, desalted using C18 cartridges and vacuum-dried. The fractions were reconstituted in 0.1% formic acid and loaded onto a reverse-phase trap column (100 μm × 2 cm) connected to an analytical column (length, 10 cm long; inner diameter, 75 μm; resin, 3 μm). The peptides were separated using a linear gradient of buffer B (84% acetonitrile and 0.1% formic acid) at a constant flow rate of 300 nL/min and analyzed using a Q Exactive mass spectrometer (Thermo Fisher Scientific Inc., Waltham, MA, United States) coupled to an Easy-nLC™ system for 60–90 min.

#### Data processing and analysis

Proteins were identified and quantified using the MASCOT engine provided by the Proteome Discoverer 1.4 software. Proteins that were differentially expressed between the two groups were screened with a fold change >1.2 and *P* < 0.05 calculated using *t*-tests. Protein subcellular localization was predicted using the utilizing CELLO software (22). The protein domain signature was predicted using the InterProScan software (23). Protein domain enrichment was analyzed using Fisher exact tests, and domains with FDR < 0.05 were considered significant. Protein functions were analyzed using Gene Ontology (GO) annotations and KEGG pathways, with *P* < 0.05 indicating significant enrichment. Protein–protein interactions (PPI) with a confidence score of 0.9 were explored using the STRING database and we constructed a PPI network that was visualized using Cytoscape v. 3.6.1 (24). Modules in the PPI network were analyzed using the Cytoscape plug-in MCODE. Significant modules were selected with > 5 nodes and scores > 4. The KEGG pathways enriched with the proteins in the significant modules were investigated using clusterProfiler v. 3.8.1 in R (25).

### Integrated metabolomic and proteomic analyses

Co-expressed pairs of differential metabolites and proteins were explored using the Spearman correlation coefficient (r) and those with adjusted *P* < 0.05, and | *r*| > 0.3 were subsequently analyzed. We investigated the pathways involved in differential metabolites and proteins using enrichment analysis using the IMPaLA tool (26), with pathway source setting as the KEGG database, num_overlapping_metabolites/genes > 0 and p_joint < 0.05. A metabolite-protein-pathway network was then constructed by integrating co-expressed pairs and common pathways and visualized using Cytoscape v. 3.6.1.

### Western blotting

Key differentially expressed proteins in the integrated metabolomic and proteomic analyses were validated using western blotting. In brief, cardiac valve samples were ground and lysed in RIPA lysis buffer containing protease inhibitor phenylmethylsulfonyl fluoride (PMSF), and protein concentrations were determined using a BCA Protein Assay kit. Proteins were separated on 10% polyacrylamide gels and transferred onto PVDF membranes. Non-specific antigen binding on the membranes was blocked using 5% skim milk in phosphate-buffered saline (PBS) for 1–2 h, and then washed three times using PBS-T buffer (1 × PBS + 1 mL of Tween-20). The membranes were incubated with anti-CaMKII delta (ab181052), anti-ectonucleotide pyrophosphatase/phosphodiesterase 1 (ENPP1)/PC-1 (ab223268), and anti-ATP Binding Cassette Subfamily A Member 8 (ABCA8) ABCA8 (ab230896) primary antibodies (all from Abcam, Cambridge, United Kingdom) at 4°C overnight, followed by HRP-conjugated secondary antibody. The membranes were washed with PBS-T buffer, and immunoreactive signals were detected using an ECL system (MilliporeSigma, Burlington, MA, United States). Gray values of protein bands were analyzed using the ImageJ software.

### Statistical analysis

Data are presented as means ± standard deviation and were analyzed using SPSS 21.0 (IBM Corp., Armonk, NY, United States). Between-group comparisons were analyzed using *t*-tests. Statistical significance was set at *P* < 0.05.

## Results

### Evaluation of cardiac valve samples

**FIGURE 1:**
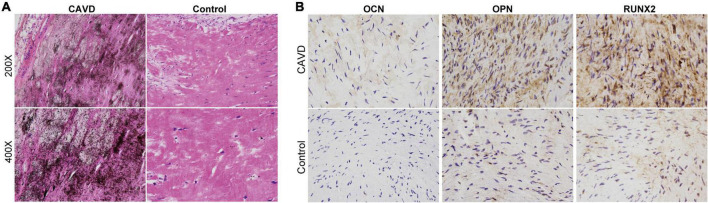
Histological evaluation of calcium deposition and osteogenic markers in cardiac valves. Representative images of panels **(A)** von Kossa staining to evaluate calcium deposition and **(B)** immunohistochemical staining of osteogenic markers in cardiac valve samples.

We histologically evaluated calcium deposition and collagen fibers in the cardiac valve samples. Figure 1A shows obvious calcium deposition and fibrosis in the samples from the CAVD compared with the controls. The immunohistochemical findings showed that expression of the osteogenic markers RUNX2, OPN, and OCN was markedly increased in CAVD, compared with that in the controls, indicating osteogenic differentiation in aortic valves with CAVD (Figure 1B).

### Metabolic profiles of heart valves in calcific aortic valve disease

Metabolites were profiled in the cardiac valve samples. After data preprocessing and batch normalization, 11,318 and 6,841 precursor molecules were obtained in the positive and negative ion modes, respectively. The PCA results showed good repeatability of the QC samples, indicating no obvious deviations in the analytical system. The proportion of characteristic peaks with RSD < 30% reached 70% in the QC samples, indicating that the quality of the data was sufficient for analysis (Supplementary Figure 2).

**FIGURE 2:**
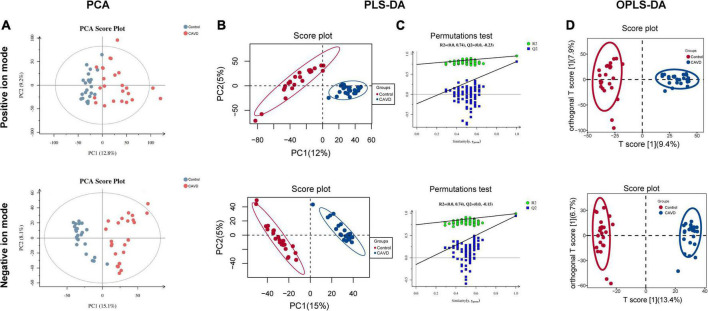
Metabolic profile changes in heart valve with calcific aortic valve disease. Principal component analysis **(A)** and PLS-DA **(B)** score plots; permutation test of PLS-DA model **(C)**. OPLS-DA score of positive and negative ion metabolites plots **(D)**. OPLS-DA, orthogonal projections to latent structures discriminant analysis; PCA, principal component analysis; PLS-DA, partial least squares discriminant analysis.

**FIGURE 3:**
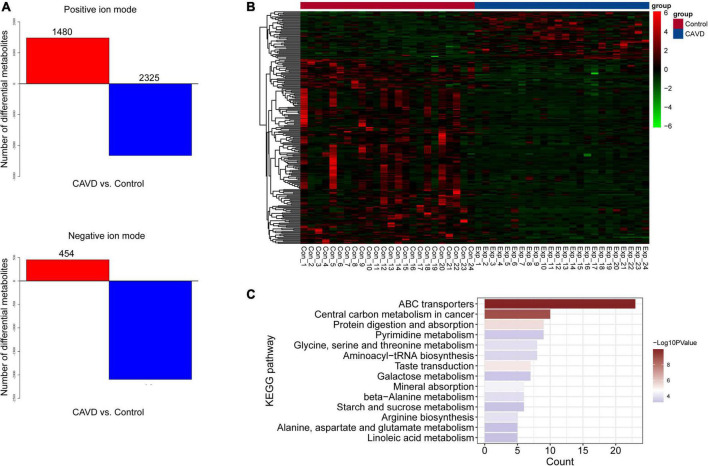
Differential metabolites and identification. **(A)** Numbers of differential positive and negative ion metabolites. Red and blue, upregulated and downregulated metabolites, respectively. **(B)** Heatmap of 229 differential metabolites. **(C)** Significantly enriched KEGG pathways of differential metabolites.

The results of PCA score plots of positive and negative ions showed that the CAVD and control sample groups tended to cluster together internally (Figure 2A), indicating a similar composition and concentration of metabolites within the same group. The PLS-DA findings showed that samples in the two groups were completely separated (Figure 2B), indicating significant differences in the metabolites. A permutation test of the PLS-DA model revealed no overfitting (Figure 2C). Variations and their contributions (Figure 2D) were also evaluated using OPLS-DA, and VIP values were calculated in the OPLS-DA model to screen differential metabolites between the two groups. In the positive ion mode, 1,480 and 2,325 metabolites were respectively upregulated and downregulated, and in the negative ion mode, 454 and 2,098 were respectively upregulated and downregulated in CAVD samples compared with the controls (Figure 3A). These findings indicated that the metabolic profile drastically changed in the cardiac valves of patients with AS.

### Metabolite identification and pathway enrichment

We identified 229 metabolites by matching with databases of known metabolites, including 170 and 59 differential positive-and negative-ion types (Supplementary Table 1). The heatmap for the 229 metabolites suggested that their profiles differed sufficiently to distinguish the two groups of samples (Figure 3B). We investigated the pathway enriched by these metabolites, and found that 14 KEGG pathways were significantly enriched, including those of ABC transporters, protein digestion, and absorption (Figure 3C). The enriched metabolic pathways comprised pyrimidine, glycine, serine, threonine, galactose, beta-alanine, starch, sucrose, alanine, aspartate, glutamate, and linoleic acid.

### Proteomic analysis

**FIGURE 4:**
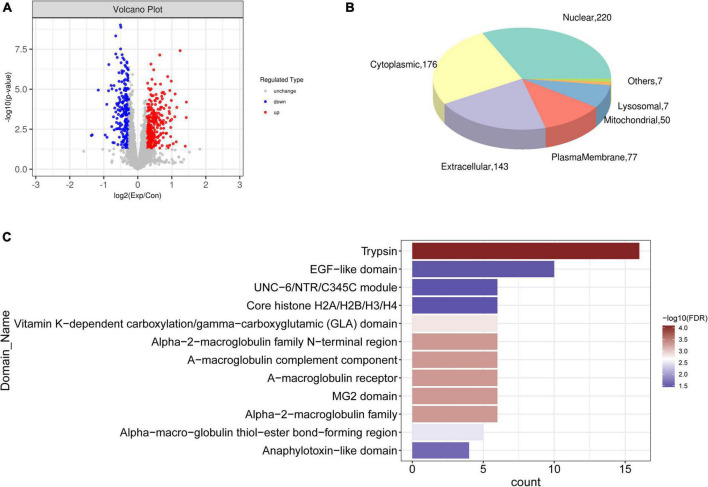
Differentially expressed proteins. **(A)** Volcano plot of differentially expressed proteins between CAVD and controls; blue and red dots, downregulated and upregulated proteins, respectively. **(B)** Pie chart of subcellular localization of differential proteins. **(C)** Significantly enriched domains of differential proteins.

Proteomic analysis identified 40,619 peptides from all samples, of which 37,067 were unique. Among 5,658 identified proteins (Supplementary Table 2), 3,649 were quantifiable (intensity value found in over 50% of the biological replicates in at least one group). Differential expression of the quantified proteins was analyzed, and 549 differential proteins were screened, including 321 and 228 that were respectively upregulated and downregulated (Figure 4A). Subcellular localization of the 549 differentially expressed proteins was predicted (Figure 4B), and most were localized in the nuclear, cytoplasmic, extracellular, and plasma membranes. Among them, 428 with unique subcellular localization comprised 55 plasma membrane, 96 cytoplasmic, 103 extracellular, 152 nuclear, 14 mitochondrial, three lysosomal, three endoplasmic reticulum protein, as well as one cytoskeletal protein and one Golgi protein. The remaining 111 proteins had two or more subcellular locations (Supplementary Table 3). These proteins were significantly enriched in trypsin, EGF, the UNC−6/NTR/C345C module, core histone H2A/H2B/H3/H4, and vitamin K-dependent carboxylation/gamma-carboxylation (GLA) domains (Figure 4C).

### Functional enrichment and protein–protein interactions network

**FIGURE 5:**
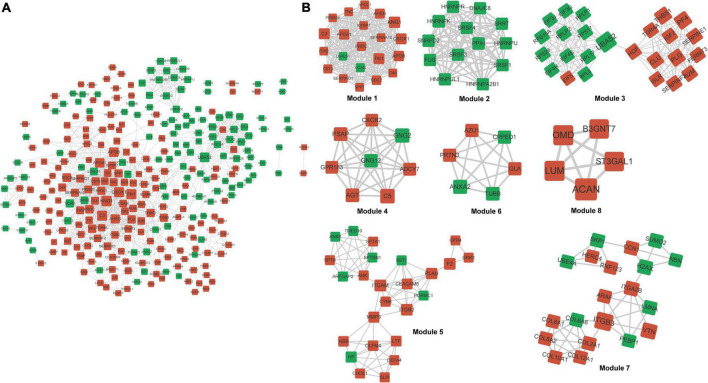
Protein–protein interaction network and modules. **(A)** Differential PPI network. Red and green nodes upregulated nodes and downregulated proteins, respectively. **(B)** Significant modules identified from PPI network. PPI, protein–protein interaction.

Gene Ontology annotations and KEGG pathways were enriched with differentially expressed proteins. Supplementary Figure 3 shows that these proteins were significantly implicated in biological processes, including extracellular structure organization and response to wounding, cellular component terms including extracellular region and extracellular matrix, and molecular function terms including glycosaminoglycan binding and endopeptidase regulator/peptidase inhibitor/endopeptidase inhibitor activities. The KEGG pathways such as cholesterol metabolism and complement and coagulation cascades were enriched. Interactions among these differentially expressed proteins were explored using PPI, and a network of 300 proteins involving 1,121 interactions was visualized (Figure 5A). Kininogen 1 (KNG1) was identified as a hub protein based on its highest degree in the network. Eight significant functional modules were identified in the PPI network (Figure 5B). Proteins in different modules were also involved in different KEGG pathways. For example, proteins in module 7 were implicated in protein digestion, absorption, and ECM-receptor interaction. Those in module 1 were implicated in vitamin/fat digestion and absorption, cholesterol metabolism, and complement and coagulation cascade pathways. Those in module 4 were enriched in multiple pathways, such as the chemokine, relaxin, and phospholipase D signaling pathways, as well as glutamatergic synapses. Regulation of the actin cytoskeleton, leukocyte transendothelial migration, and neutrophil extracellular trap formation were enriched with proteins in module 5 (Supplementary Table 4).

### Co-expression of differential metabolites and differential proteins

Associations between differential metabolites and proteins were explored using Spearman correlations. We screened 21,591 co-expressed pairs involving 216 metabolites and 546 proteins with adjusted *P* < 0.05 and | *r*| > 0.3. Table 2 shows co-expressed pairs with | *r*| > 0.7. Selenium binding protein 1 (SELENBP1) significantly and positively correlated with multiple metabolites, including (< U + 00C2 >< U + 00B1 >)-metalaxyl, sinensetin, naringenin, tangeritin, naringin, isovitexin 2-O-beta-D-glucoside, and sorbitol.

**TABLE 2:** Co-expression of differential metabolites and differential proteins with absolute value of Spearman correlation coefficient more than 0.7.

Metabolite KEGG id	Metabolite name	Protein	*r*	*P*-value	Adj.p.val
C00794	Sorbitol	PLEK	−0.747	1.05E−09	2.65E−05
C00300	Creatine	PROC	−0.733	2.35E−08	2.27E−04
C00300	Creatine	TUBAL3	−0.73	2.79E−08	2.34E−04
C10190	Tangeritin	FLT1	−0.718	9.49E−09	1.61E−04
C00794	Sorbitol	COL8A2	−0.705	1.00E−07	4.26E−04
C21308	(S)-beta-Tyrosine	PIR	0.703	2.58E−08	2.32E−04
C10190	Tangeritin	NCL	0.705	1.02E−07	4.26E−04
C00794	Sorbitol	HDGFL3	0.705	2.26E−08	2.27E−04
C21308	(S)-beta-Tyrosine	DDAH2	0.708	8.78E−08	4.09E−04
C00794	Sorbitol	SKP1	0.709	8.18E−08	3.96E−04
C21308	(S)-beta-Tyrosine	CYGB	0.716	6.01E−08	3.44E−04
C09789	Naringin	SELENBP1	0.717	1.02E−08	1.61E−04
C04199	Isovitexin 2-O-beta-D-glucoside	SELENBP1	0.718	9.18E−09	1.61E−04
C00794	Sorbitol	SELENBP1	0.737	1.77E−08	2.03E−04
C00794	Sorbitol	RBP1	0.74	1.32E−08	1.65E−04
C00509	Naringenin	SELENBP1	0.742	1.18E−08	1.65E−04
C10190	Tangeritin	SELENBP1	0.757	0.00E + 00	0.00E + 00
C00794	Sorbitol	ITGB4	0.757	4.63E−10	1.45E−05
C10186	Sinensetin	SELENBP1	0.771	0.00E + 00	0.00E + 00
C10947	(<U + 00C2 >< U + 00B1 >)-Metalaxyl	SELENBP1	0.785	0.00E + 00	0.00E + 00

### Metabolite-protein-pathway network

**FIGURE 6:**
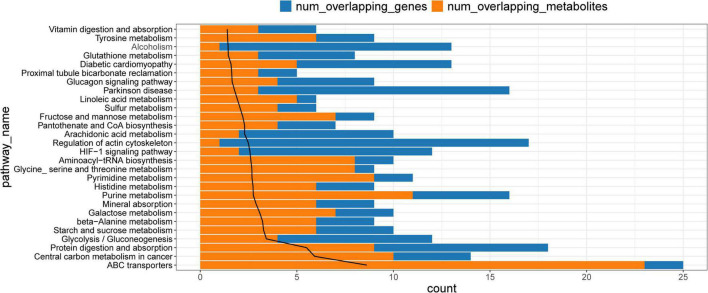
Kyoto Encyclopedia of Genes and Genomes pathways enriched by differential metabolites and proteins. KEGG, Kyoto Encyclopedia of Genes and Genomes.

**FIGURE 7:**
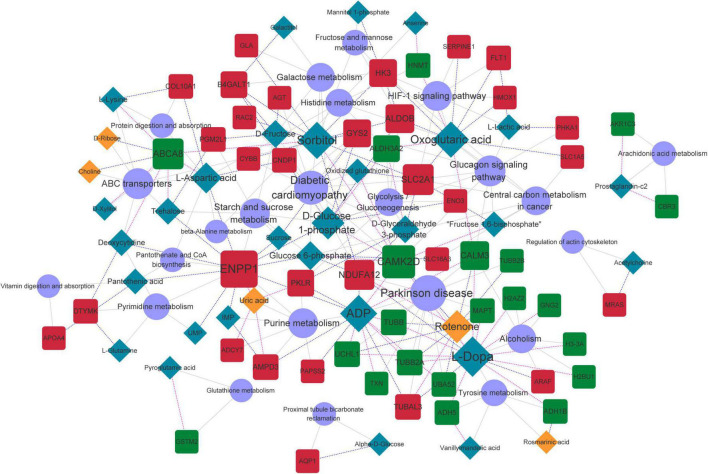
Metabolite-protein-pathway network. Orange and blue rhombus nodes, upregulated and downregulated metabolites. Red and blue square nodes, upregulated and downregulated proteins. Purple circle nodes, represent KEGG pathways. Node size represents degree of nodes in network. KEGG, Kyoto Encyclopedia of Genes and Genomes.

**FIGURE 8:**
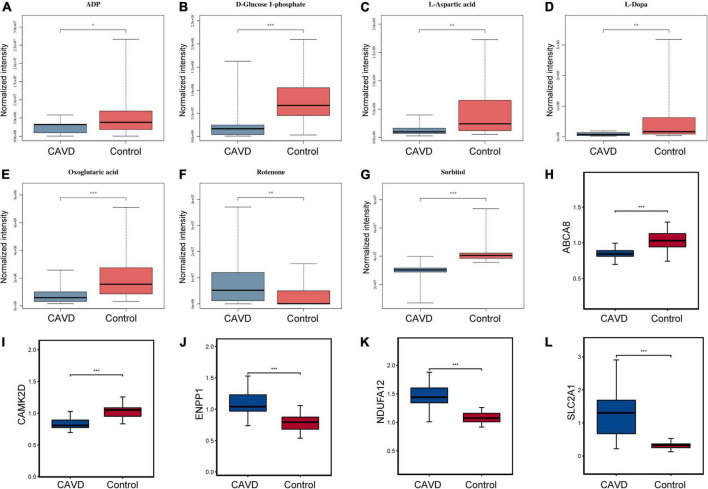
Levels of hub metabolites and proteins. Boxplots show levels of panels **(A–G)** hub metabolites ADP, D-glucose 1-phosphate, L-aspartic acid, L-dopa, oxoglutaric acid, rotenone, and sorbitol and **(H–L)** hub proteins ENPP1, CAMK2D, ABCA8, SLC2A1, and NDUFA12 in CAVD and control samples. *P < 0.05; **P < 0.01; ***P < 0.001 vs. control. ABCA8, ATP Binding Cassette Subfamily A Member 8, CAMK2D, calcium2 + /calmodulin-dependent protein kinase II delta; ENPP1, ectonucleotide pyrophosphatase/phosphodiesterase 1NDUFA12, NADH:ubiquinone oxidoreductase subunit A12; SLC2A, solute carrier family 2 member 1.

Common pathways enriched by differential metabolites and proteins were analyzed using the IMPaLA tool. We found 28 significantly enriched KEGG pathways (Figure 6) that included those of ABC transporters, protein digestion and absorption, and glycolysis/gluconeogenesis, starch and sucrose, beta-alanine, pyrimidine, purine, histidine, and galactose metabolic pathways. These pathways and co-expression pairs were integrated to construct a metabolite-protein pathway network. Figure 7 shows that the network consisted of 34 metabolites, 52 proteins, and 23 pathways. The degree of connection of each node in the network was further analyzed to screen hub metabolites, proteins, and their involved pathways. The top 20 nodes included seven hub metabolites (L-Dopa, sorbitol, ADP, oxoglutaric acid, d-glucose 1-phosphate, rotenone, and L-aspartic acid), five hub proteins ENPP1, calcium^2+^/calmodulin-dependent protein kinase II delta (CAMK2D), ATP Binding Cassette Subfamily A Member 8 (ABCA8), Solute Carrier Family 2 Member 1 (SLC2A1), and NADH:ubiquinone oxidoreductase subunit a12 (NDUFA12), and eight pathways (ABC transporters, starch and sucrose metabolism, hypoxia-inducible factor 1-alpha (HIF-1) signaling pathway, and purine metabolism). The hub metabolites were all downregulated in CAVD samples compared with controls, except for rotenone (Figures 8A–G). Hub proteins ENPP1, SLC2A1, and NDUFA12 were upregulated, whereas CAMK2D and ABCA8 were downregulated in CAVD compared with controls (Figures 8H–L).

### Validation of hub proteins using western blotting

The western blotting results validated the expression of the hub proteins ENPP1, CAMK2D, and ABCA8. Consistent with the proteomics findings, ENPP1 was significantly upregulated (*P* < 0.05), whereas CAMK2D and ABCA8 protein expression were significantly downregulated (*P* < 0.05) in CAVD compared with controls (Supplementary Figure 1).

## Discussion

This is the first analysis of cardiac valve samples from patients with CAVD using integrated metabolomics and proteomics as far as we can ascertain. Although the molecular mechanisms and targets for CAVD have been analyzed using omics (27, 28), definitive biomarkers or landscapes of molecular mechanisms could not be elucidated. This may be attributed to the pathological processes of CAVD and physical and chemical activities of biological samples that are too complex and diverse for a single omics analysis.

The heart is the organ of choice for metabolic studies, as it consumes numerous substrates to produce ATP for contraction and to maintain biosynthetic reactions for tissue remodeling and repair (29). Metabolism regulates cardiac structure and function. Patients with severe AS often present with cardiac remodeling, caused by changes in metabolism. The present study identified 229 differential metabolites among which, 2-aminophenol, hydroxykynurenine, erythritol, carnosine, and choline were the top five between AS and control groups. The primary pathway of tryptophan catabolism in most mammalian cells is that of kynurenine, which functions as an inflammatory sensor and modulator during vascular disease development by generating bioactive catabolites such as 3-hydroxykynurenine. Such bioactive catabolites are potential targets for the initiation and progression of aortic diseases (30, 31). Erythritol is a polyol that can improve small-vessel endothelial function and reduce central aortic stiffness (32). Carnosine is a decisive factor in the formation of atherosclerotic lesions, and it can repress atherogenesis by promoting the removal of aldehydes derived from oxidized lipids (33). Based on a calcification model of vascular smooth muscle cells (VSMCs), Huang et al. (34) reveals that carnosine can attenuate calcium deposition in a dose-dependent way by reducing expression of osteogenesis-related proteins RUNX2 and BMP2. Compared with normal controls, plasma choline levels are significantly increased in patients with AS, as well as in patients whose aortic valves had neovascularization, calcification, metaplastic ossification, and more severe tissue remodeling. Plasma choline also independently correlated with aortic peak flow velocity (35, 36). Cardiovascular parameters can be altered by intraperitoneal administration of cytidine-5’-diphosphocholine (CDP-choline) and its metabolites (37). Choline-derived metabolite trimethylamine N-oxide, a gut microbiota-generated metabolite, is linked to the development of cardiovascular diseases; and it can induce osteogenic differentiation of aortic valve interstitial cells *in vitro* and promote aortic valve lesions *in vivo* (38). In addition, several proteins involving choline are identified and/or quantified in proteomic analysis, including phospholipase autotoxin (ENPP2), lysophosphatidic acid receptor 1 (LPAR1) and Acyl-protein thioesterase 1 (LYPLA1) and so on. ENPP2 is a phospholipase D that associates with Lp(a) and cleaves lysophosphatidyl choline (LysoPC) to generate both choline and lysophosphatidic acid (LysoPA) — the latter, an osteogenic stimulus for valve interstitial cells *via* the LPAR1 receptor (39, 40). LYPLA1, also named lysophospholipase 1, contributes to the production of LysoPA during blood coagulation by recognizing and cleaving plasma phospholipids to generate lysophospholipids which in turn act as substrates for ENPP2 to produce LysoPA (41). These findings suggested that hydroxykynurenine, erythritol, carnosine, and choline could be potential targets or biomarkers for AS.

Proteomic analysis identified 549 differential proteins, most of which were localized in nuclear, cytoplasmic, extracellular, and plasma membranes. Kininogen 1 was identified as a hub protein based on its highest degree in the PPI network. Angiotensin-converting enzyme (ACE) inhibitors might confer survival benefits in AS by regulating left ventricular remodeling and myocardial fibrosis (42, 43). Kininogen 1 is important in ACE-related pathways, and variations in KNG1 correlate with decreased risk of sudden cardiac arrest (44). Abundant KNG1 expression might aggravate mitochondrial damage and oxidative stress in the heart (45). Eight significant functional modules were identified in the PPI network, and the proteins in these modules were involved in various KEGG pathways. For example, proteins in module 1 were involved in vitamin/fat digestion, absorption, and cholesterol metabolism. Low levels of vitamin D have been linked to the pathogenesis of atherosclerosis as well as vascular calcification that significantly correlate with left ventricular wall thickness, indicating the role of vitamin D in regulating hypertrophic remodeling (46). Dyslipidemia is an independent risk factor for cardiovascular disease. Lipid-lowering therapy can reduce the morbidity and mortality associated with cardiovascular diseases (47). Increased lipid can be deposited at sites of valve damage, causing calcification of surrounding tissues. Lipid-lowering statin drugs primarily inhibit endogenous cholesterol synthesis of the rate-limiting enzyme 3-hydroxy-3-methylglutaryl coenzyme A (HMG-COA) reductase through competitive inhibition, and block the intracellular hydroxymevalonate metabolic pathway. This would reduce intracellular cholesterol synthesis, stimulate the number and activity of low-density lipoprotein (LDL) receptors, and improve lipid levels (48, 49).

We found that SELENBP1 significantly and positively correlated with multiple metabolites. This protein is a marker of white adipocytes and intracellular lipid accumulation that mediates lipid metabolism and adipogenesis (50, 51). Considering the importance of lipid levels, we concluded that SELENBP1 might be a crucial target in AS. We constructed a metabolite-protein-pathway network, and the top 20 nodes included seven hub metabolites, including L-Dopa, sorbitol, ADP, oxoglutaric acid, d-glucose 1-phosphate, rotenone, and L-aspartic acid. Of which, all metabolite was down-regulated in CAVD in addition to rotenone. Rotenone is a natural product derived from the stems/roots of some plants, such as Lonchocarpus, Derris and Mundulea plant species; which is a widely used organic pesticide that inhibits complex I of the mitochondrial electron transport system (52). Despite the well-known neurotoxic effect, rotenone can attenuate the fibrotic response in chronic obstructive uropathy (53), attenuate tubulointerstitial fibrosis and tubular damage induced by unilateral renal ischemia and reperfusion (54), and show anti-tumor effect (55, 56). Few studies report the effects of low-dose rotenone exposure on the heart. Zhan et al. (57) investigates the cardiotoxicity of rotenone in rats, and indicates that rotenone can increase the risk of ventricular arrhythmias and lead to electrical and structural cardiac remodeling. There is no evidence that rotenone increases the risk of CAVD or that it affects valve aortic interstitial cells calcification *in vitro* so far. More investigations are needed to explore the effects of low-dose rotenone exposure on the heart.

In the metabolite-protein-pathway network, ENPP1, CAMK2D, and ABCA8 were identified as hub proteins. We confirmed the expression of these proteins using western blotting. Calcium^2+^/calmodulin-dependent protein kinase II delta belongs to the CaMKII family, and it acts as a messenger in cardiomyocytes. Splice variants in the nucleus and cytoplasm are usually considered mediators of contractile function, arrhythmias, and Ca^2+^ handling (58). ENPP1 cleaves ATP to generate extracellular inorganic pyrophosphate (PPi), an endogenous mineralization inhibitor, and a deficiency of this enzyme leads to arterial calcification. Recombinant human (rh)ENPP1-Fc might have potential in preventing and treat AS in generalized arterial calcification in infancy (59, 60). We found that CAMK2D interacted with downregulated metabolites, oxidized glutathione, glucose 6-phosphate, D-glucose 1-phosphate, and ADP, and that ENPP1 similarly interacted with glucose 6-phosphate, D-glucose 1-phosphate, and ADP metabolites. The energy supply mechanism for the interconversion of ATP and ADP is common in biology. The heart consumes several substrates to produce ATP for contraction and to maintain biosynthetic reactions for tissue remodeling and repair (29). Oxidative stress, which regulates the degradation and remodeling of the aorta, is a major molecular mediator in the evolution of AS. High-density lipoprotein cholesterol has been linked to oxidative stress in patients with AS (61). Glucose 6-phosphate dehydrogenase activity determines cytoplasmic NADPH to NADP ratios and promotes replenishment of the antioxidant glutathione system. A loss of function can increase oxidative stress. Mice deficient in glucose 6-phosphate dehydrogenase are susceptible to cardiovascular disease and ventricular dilation in response to myocardial infarction or pressure overload-induced heart failure (62, 63). High-density lipoprotein (HDL) levels regulated by ABCA8 promotes cholesterol efflux (64). We found that ABCA8 interacted with the upregulated metabolite choline. Plasma choline levels are significantly increased in patients with AS, and in patients whose aortic valves exhibited neovascularization, calcification, and metaplastic ossification, as well as more severe tissue remodeling. Plasma choline also independently correlates with aortic peak flow velocity (35, 36). These findings confirmed the importance of ENPP1, CAMK2D, and ABCA8 in the development of CAVD.

Despite these findings, there also remained several limitations in this study. (1) We preliminarily identified several metabolites and proteins as potential biomarkers. However, their predictive value in CAVD has not been investigated. For example, key differential metabolites should be evaluated by receiver operator characteristic curve to investigate their diagnostic value in CAVD. (2) The biological functions (e.g., regulating calcification of valve aortic interstitial cells) of hub proteins in the development of CAVD are unclear, and it should be confirmed by a series of functional experiments. (3) There was significant differences in terms of age and diabetes between CAVD and control groups. This may bring some influences to the results. Studies have demonstrated that compared to than non-diabetic patients, patients diabetes showed a significantly elevated risk of CAVD, a significantly greater propensity for developing rapidly progressive AS, and a high degree of calcification in diseased aortic valves (65, 66). This may explain the differences of diabetes between CAVD and control groups.

## Conclusion

This is the first study to uncover changes in metabolic and protein profiles in cardiac valve samples of patients with CAVD using an integrated metabolomics and proteomics approach. We identified 229 and 549 differentially expressed metabolites and proteins, respectively. We considered that the significantly differential metabolites (hydroxykynurenine, erythritol, carnosine, and choline) and proteins (SELENBP1, ENPP1, CAMK2D, and ABCA8) may be potential targets or novel biomarkers of CAVD and thus serve as the foundation for further investigation of CAVD.

## Data availability statement

The datasets generated for this study can be found in the iProx repository: https://www.iprox.cn//page/project.html?id=IPX0005050000, accession number: IPX 0005050001.

## Ethics statement

The studies involving human participants were reviewed and approved by the Medical Ethics Committee at our hospital approved the study (IRB protocol number: IRB-SOP-016F-001-02-2022LW-008). The patients/participants provided their written informed consent to participate in this study.

## Author contributions

BF and NJ contributed to the conception and design. ZG contributed to the administrative support. BF, LW, QW, and NJ contributed to the provision of study materials or patients. NJ did the collection and assembly of data. BF, JW, and MX did the data analysis and interpretation. All authors wrote and finally approved the manuscript.

## Supplementary material

Supplementary Figure S1 Western blots and protein quantitation in CAVD and control samples. CAVD, calcific aortic valve disease. **P* < 0.05.

Supplementary Figure S2 Quality control analysis of untargeted metabolomics. PCA score plot and quality assurance of positive and negative ions, respectively. PCA, principal component analysis.

Supplementary Figure S3 Functional enrichment analysis of differential proteins. Bubble diagram of significantly enriched Gene Ontology annotation, including biological process, cellular component, molecular function terms, and KEGG pathways of differential proteins. KEGG, Kyoto Encyclopedia of Genes and Genomes.
