# Supplementary figures and images for: Integrated proteomic and metabolomic profile analyses of cardiac valves revealed molecular mechanisms and targets in calcific aortic valve disease
Source: Front Cardiovasc Med. 2022 Oct 13;9:944521. doi: 10.3389/fcvm.2022.944521 (PMC9606238; doi:10.3389/fcvm.2022.944521)

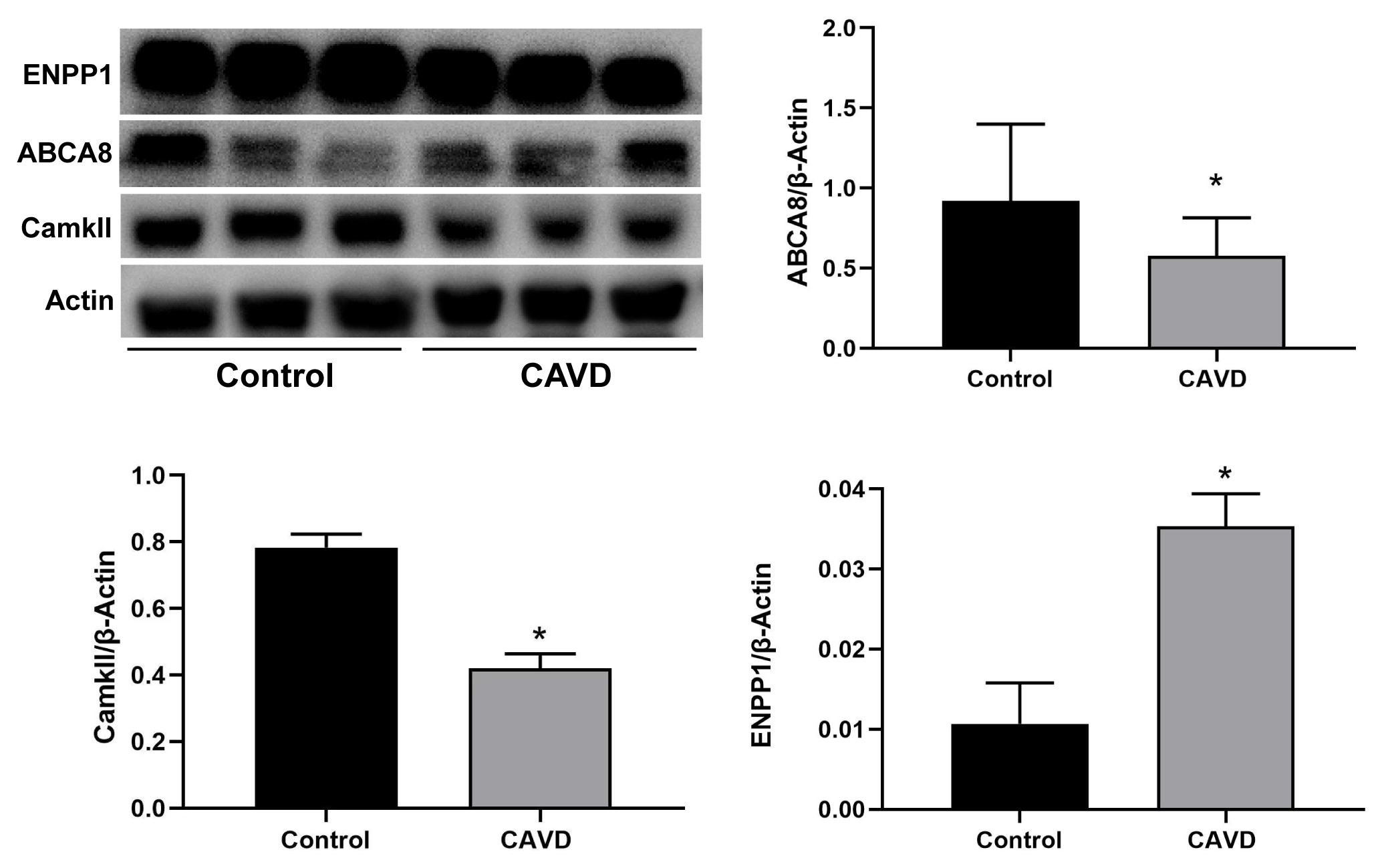

Supplement: Supplementary Figure S1 — Western blots and protein quantitation in CAVD and control samples. CAVD, calcific aortic valve disease. *P < 0.05. [file Image_1.TIF]

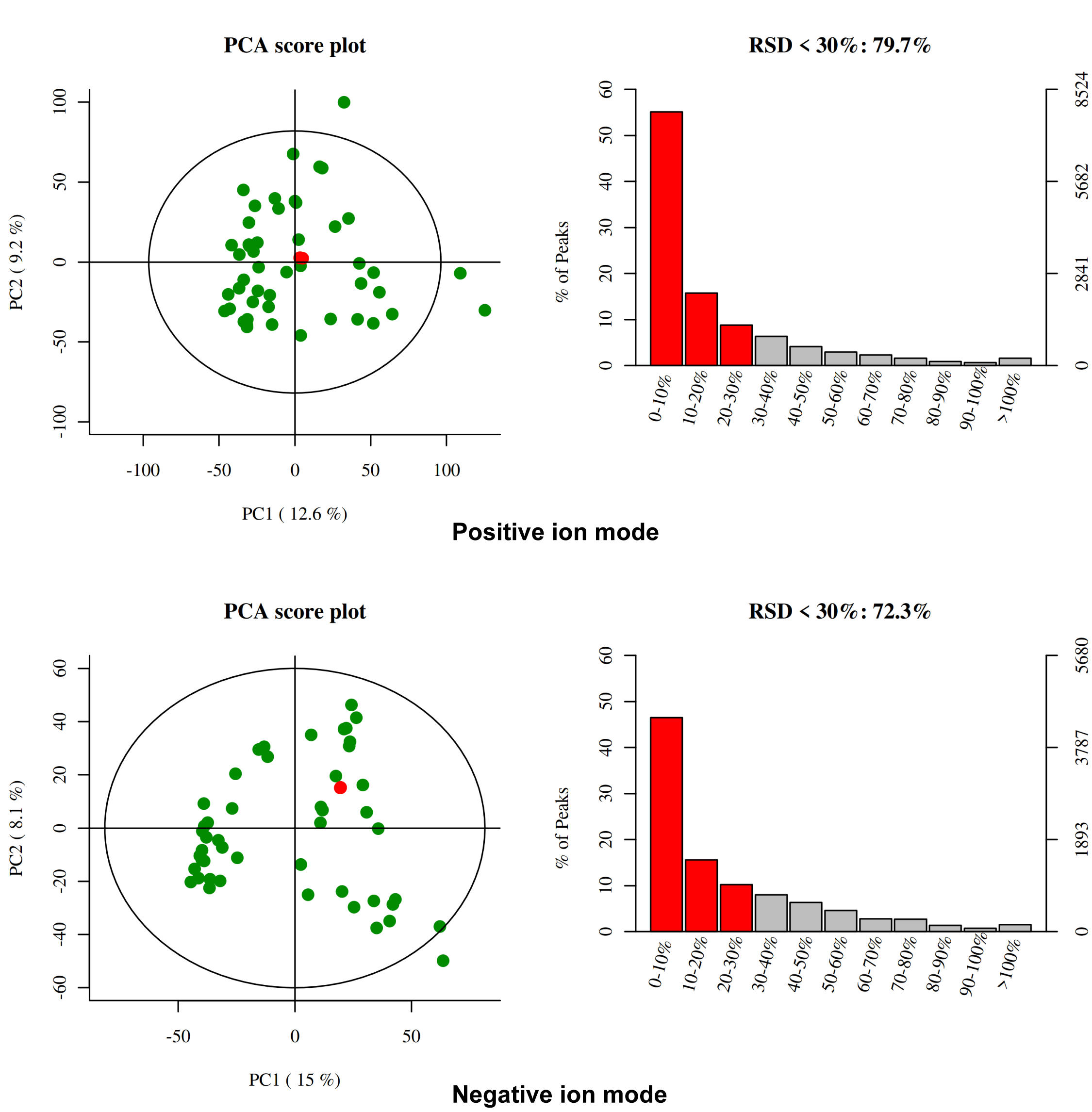

Supplement: Supplementary Figure S2 — Quality control analysis of untargeted metabolomics. PCA score plot and quality assurance of positive and negative ions, respectively. PCA, principal component analysis. [file Image_2.TIF]

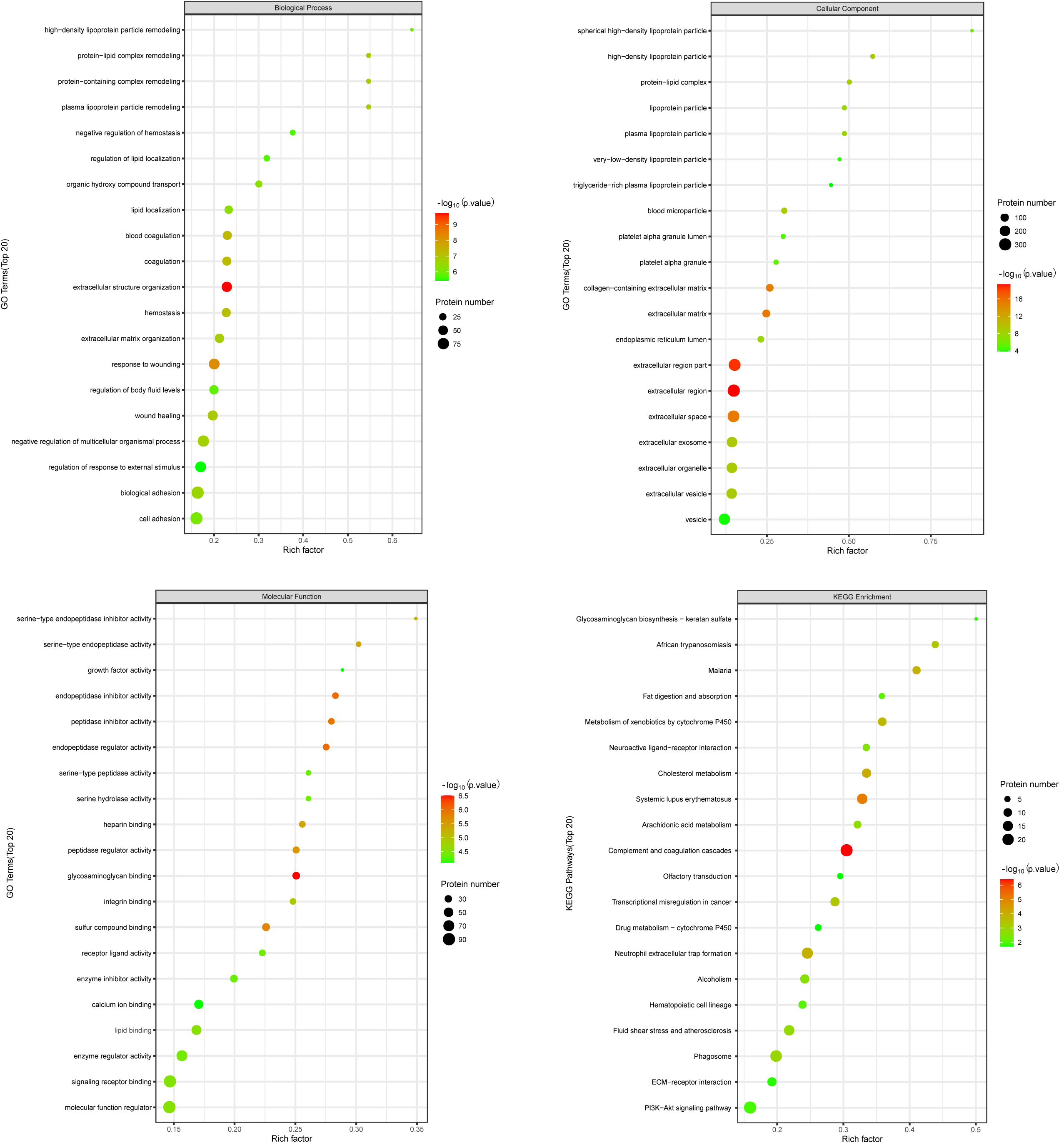

Supplement: Supplementary Figure S3 — Functional enrichment analysis of differential proteins. Bubble diagram of significantly enriched Gene Ontology annotation, including biological process, cellular component, molecular function terms, and KEGG pathways of differential proteins. KEGG, Kyoto Encyclopedia of Genes and Genomes. [file Image_3.TIF]
