# Supplementary material for: Integrated proteomic and metabolomic profile analyses of cardiac valves revealed molecular mechanisms and targets in calcific aortic valve disease
Source: Front Cardiovasc Med. 2022 Oct 13;9:944521. doi: 10.3389/fcvm.2022.944521 (PMC9606238; doi:10.3389/fcvm.2022.944521)
Supplement: Supplementary file 4 [file Table_1.DOCX]

**Table 1.** **Clinical characteristics of CAVD patients and control individuals.**

|  | CAVD  (n=24) | Control  (n=24) | *P* value |
| --- | --- | --- | --- |
| Age (years) | 66.5±7.8 | 59.5±12.0 | <0.05 |
| Male, n (%) | 12, (50.0%) | 14, (58.3%) | 0.5623 |
| Body mass index | 23.3±3.0 | 23.0±3.7 | 0.8515 |
| LVEF, % | 55.5±8.0 | 52.3±9.8 | 0.2525 |
| Medical history, n (%) |  |  |  |
| Hypotension | 11, (45.8%) | 16, (66.7%) | 0.2443 |
| Diabetes | 5, (20.8%) | 0, (0%) | <0.05 |
| CHD | 15, (62.5%) | 9, (37.5%) | 0.0833 |
| Preoperative Medication, n (%) |  |  |  |
| ACEI | 1, (4.1%) | 5, (20.8%) | 0.0809 |
| BB | 18, (75.0%) | 14, (63.6%) | 0.4028 |
| CCB | 8, (33.3%) | 5, (20.8%) | 0.3299 |
| Metabolic data |  |  |  |
| HDL (mmol/L) | 1.04±0.36 | 1.09±0.23 | 0.64 |
| LDL (mmol/L) | 2.89±1.17 | 2.36±0.67 | 0.07 |
| VLDL ( mmol/L) | 0.21 (0.20) | 0.29 (0.24) | 0.26 |
| Triglyceride (mmol/L) | 1.02 (0.59) | 1.25 (1.07) | 0.44 |
| Lipoprotein A (nmol/L) | 37.40 (50.25) | 45.25 (58.20) | 0.89 |
| Apolipoprotein A (g/L) | 1.22±0.24 | 1.25±0.18 | 0.65 |
| Apolipoprotein B (g/L) | 0.99±0.29 | 0.83±0.20 | 0.04 |

Note: Data was presented as mean ± standard deviation or n (%) or median (interquartile range); LVEF, left ventricular ejection fraction; CHD, congenital heart disease; ACEI, angiotensin-converting enzyme inhibitors; BB, beta-blocker; CCB, calcium channel blockers; HDL, high-density lipoprotein; LDL, low-density lipoprotein; VLDL, very low-density lipoprotein.

**Table 2. Co-expression of differential metabolites and differential proteins with absolute value of Spearman correlation coefficient more than 0.7.**

| **Metabolite KEGG ID** | **Metabolite name** | **Protein** | **r** | **P value** | **adj.p.val** |
| --- | --- | --- | --- | --- | --- |
| C00794 | Sorbitol | PLEK | -0.747 | 1.05E-09 | 2.65E-05 |
| C00300 | Creatine | PROC | -0.733 | 2.35E-08 | 2.27E-04 |
| C00300 | Creatine | TUBAL3 | -0.73 | 2.79E-08 | 2.34E-04 |
| C10190 | Tangeritin | FLT1 | -0.718 | 9.49E-09 | 1.61E-04 |
| C00794 | Sorbitol | COL8A2 | -0.705 | 1.00E-07 | 4.26E-04 |
| C21308 | (S)-beta-Tyrosine | PIR | 0.703 | 2.58E-08 | 2.32E-04 |
| C10190 | Tangeritin | NCL | 0.705 | 1.02E-07 | 4.26E-04 |
| C00794 | Sorbitol | HDGFL3 | 0.705 | 2.26E-08 | 2.27E-04 |
| C21308 | (S)-beta-Tyrosine | DDAH2 | 0.708 | 8.78E-08 | 4.09E-04 |
| C00794 | Sorbitol | SKP1 | 0.709 | 8.18E-08 | 3.96E-04 |
| C21308 | (S)-beta-Tyrosine | CYGB | 0.716 | 6.01E-08 | 3.44E-04 |
| C09789 | Naringin | SELENBP1 | 0.717 | 1.02E-08 | 1.61E-04 |
| C04199 | Isovitexin 2-O-beta-D-glucoside | SELENBP1 | 0.718 | 9.18E-09 | 1.61E-04 |
| C00794 | Sorbitol | SELENBP1 | 0.737 | 1.77E-08 | 2.03E-04 |
| C00794 | Sorbitol | RBP1 | 0.74 | 1.32E-08 | 1.65E-04 |
| C00509 | Naringenin | SELENBP1 | 0.742 | 1.18E-08 | 1.65E-04 |
| C10190 | Tangeritin | SELENBP1 | 0.757 | 0.00E+00 | 0.00E+00 |
| C00794 | Sorbitol | ITGB4 | 0.757 | 4.63E-10 | 1.45E-05 |
| C10186 | Sinensetin | SELENBP1 | 0.771 | 0.00E+00 | 0.00E+00 |
| C10947 | (<U+00C2><U+00B1>)-Metalaxyl | SELENBP1 | 0.785 | 0.00E+00 | 0.00E+00 |
